# Effectiveness of postoperative progressive relaxation exercises on pain, anxiety, and physiological parameters after emergency abdominal surgery: a randomized controlled trial

**DOI:** 10.1007/s11845-026-04357-4

**Published:** 2026-04-02

**Authors:** Volkan Gökmen, Tuluha Ayoğlu

**Affiliations:** 1 Department of Nursing, Faculty of Health Sciences, Ağrı İbrahim Çeçen University, Ağrı, Türkiye; 2 Department of Nursing, Istanbul University–Cerrahpaşa, İstanbul, Türkiye

**Keywords:** Anxiety, Emergency surgery, Postoperative pain, Progressive relaxation exercises, Randomised controlled trial

## Abstract

**Background:**

Emergency abdominal surgery is associated with pronounced physiological stress, severe postoperative pain, heightened anxiety, and early autonomic dysregulation. Evidence-based non-pharmacological interventions delivered exclusively during the postoperative period remain limited in emergency surgical settings.

**Aims:**

To evaluate whether postoperative Progressive Relaxation Exercises (PRE) reduce pain and anxiety and improve physiological parameters following emergency abdominal surgery, independent of analgesic consumption.

**Methods:**

This parallel-group randomized controlled trial included 70 adults undergoing emergency abdominal surgery. Participants were randomized to receive PRE plus standard postoperative care or standard care alone. PRE was delivered individually at the bedside in three 30-min sessions during the early postoperative period. Pain, anxiety, and physiological parameters were assessed using validated instruments. Analyses were performed using analysis of covariance adjusted for baseline variables, in accordance with CONSORT 2010 guidelines.

**Results:**

By postoperative day 2, the PRE group demonstrated significantly lower total pain scores compared with controls (mean difference –7.67, 95% CI –10.35 to –4.99; *p* < 0.001), with large effect sizes (Cohen’s d up to 1.51). State and trait anxiety were also significantly reduced (mean differences –7.78 and –7.52, respectively; both *p* = 0.004). At postoperative hour 6, PRE participants showed lower heart rate (–2.03 bpm), lower respiratory rate (–3.03 breaths/min), and higher oxygen saturation (+ 1.57%), indicating early autonomic stabilization. These physiological differences were transient and resolved by postoperative day 2. Analgesic consumption did not differ between groups.

**Conclusion:**

Postoperative Progressive Relaxation Exercises are a feasible, nurse-delivered intervention that significantly reduce pain and anxiety and support early physiological stabilization after emergency abdominal surgery.

## Introduction

Emergency abdominal surgery presents a uniquely intense physiological and psychological burden, characterised by abrupt clinical deterioration, limited opportunity for preoperative counselling, and pronounced sympathetic activation. Unlike elective surgical populations, patients undergoing emergency procedures enter the perioperative period with elevated catecholamine release, heightened hypothalamic–pituitary–adrenal (HPA) axis activity, increased muscle tension, and amplified nociceptive processing. These mechanisms converge to create a high-risk physiological state marked by greater postoperative pain, anxiety, and instability in cardiopulmonary parameters [1, 2].

Postoperative pain is not solely a function of tissue injury; it is profoundly shaped by emotional distress, uncertainty, and fear. Anxiety, in particular, has been shown to intensify central sensitisation, reduce pain thresholds, delay mobilisation, and increase analgesic demand through interacting neurophysiological pathways that link affective processing with nociceptive modulation [3, 4]. In high-acuity surgical contexts, this reciprocal relationship can manifest as a pain–anxiety amplification loop, whereby heightened distress augments pain perception, and escalating pain further exacerbates anxiety, thereby sustaining sympathetic overactivation and compromising physiological recovery, a mechanism well documented in mind–body intervention research [4, 5]. Progressive Relaxation Exercises (PRE) is a structured technique involving the sequential contraction and relaxation of major muscle groups that modulates autonomic arousal by enhancing parasympathetic activity, reducing muscle tension, and improving bodily awareness [6]. Through these mechanisms, PRE has been shown to attenuate stress-related neuroendocrine responses, including excessive hypothalamic–pituitary–adrenal axis activation, and to support adaptive physiological and psychological regulation in clinical populations [7, 8]. Although PRE has demonstrated beneficial effects on pain, anxiety, and physiological stress responses in elective surgical settings, oncology, chronic illness, and medical anxiety contexts, existing evidence has predominantly focused on preoperative or perioperative applications [9–11]. To date, no high-quality randomised controlled trial has rigorously evaluated the effectiveness of PRE when delivered exclusively during the early postoperative period in patients undergoing emergency abdominal surgery. This represents a critical gap in the literature, particularly given the restricted therapeutic window, heightened autonomic instability, and limited opportunity for preoperative psychological preparation that characterise emergency surgical care [2, 12].

The absence of evidence-based, scalable, and nurse-deliverable non-pharmacological interventions during the immediate postoperative phase further underscores the clinical relevance of the present study. In emergency surgery units, pharmacological pain management is frequently constrained by haemodynamic instability, organ dysfunction, or concerns related to polypharmacy, increasing the need for safe adjunctive strategies that support autonomic regulation and stress reduction [4, 13, 14]. Within this context, PRE offers a low-cost, standardisable, and bedside-applicable intervention that can be integrated into routine postoperative nursing care, with the potential to complement multimodal analgesia and Enhanced Recovery After Surgery (ERAS)–oriented pathways [10, 15]. Therefore, this randomised controlled trial was designed to evaluate the effectiveness of postoperative Progressive Relaxation Exercises when implemented during the early recovery period following emergency abdominal surgery. Specifically, the study aimed to determine whether the addition of PRE to standard postoperative care would result in reductions in postoperative pain intensity and multidimensional pain experience, decreases in both state and trait anxiety, and improvements in physiological parameters (heart rate, respiratory rate, blood pressure, oxygen saturation) associated with postoperative recovery [13–15].

By addressing these objectives within a high-risk emergency surgical population, this study seeks to generate rigorous evidence to inform the integration of nurse-led, non-pharmacological interventions into multimodal postoperative recovery pathways, including Enhanced Recovery After Surgery–aligned care models [16].

## Methods

### Study design

This study was conducted as a parallel-group, superiority randomised controlled trial in accordance with the CONSORT 2010 Statement for randomised trials [17]. The trial evaluated the effectiveness of Progressive Relaxation Exercises delivered exclusively during the early postoperative period following emergency abdominal surgery. The study protocol was prospectively registered at ClinicalTrials.gov (NCT07301073) prior to participant enrolment, and no protocol amendments were made after recruitment commenced. The design and reporting were structured to minimise selection, performance, and detection bias while ensuring transparency and reproducibility.

### Setting

The study was carried out in the General Surgery Unit of Ağrı Training and Research Hospital, a tertiary-level centre providing continuous 24-h emergency surgical services. Standard postoperative care procedures including analgesic management, mobilisation protocols, routine nursing care, and vital sign monitoring were uniformly applied across the unit to minimise inter-provider variability and contextual confounding during the study period.

### Participants

Eligible participants were adults aged 18 years or older who underwent emergency abdominal surgery requiring operative intervention within 24 h of admission. Inclusion criteria comprised being conscious and cooperative, having no hearing impairment, no cognitive impairment or neurological deficit affecting sensory perception, and being haemodynamically stable and able to participate in the intervention by postoperative hour 6. These criteria were selected to ensure patient safety and the capacity to engage reliably in the relaxation protocol while reflecting the clinical characteristics of emergency surgical populations [16].

Exclusion criteria included the presence of psychiatric disorders requiring active treatment, prior participation in structured relaxation therapy, postoperative admission to the intensive care unit, administration of sedative medications that could interfere with participation, and uncontrolled haemodynamic instability. Emergency surgery was operationally defined as a non-elective surgical procedure required within 24 h to prevent morbidity or mortality, consistent with established surgical definitions and emergency care standards [2].

### Sample size calculation

An a priori sample size calculation was performed using G*Power version 3.1 to determine the number of participants required to detect a clinically meaningful between-group difference in postoperative pain outcomes. Based on effect sizes reported in prior randomised trials evaluating relaxation-based interventions in postoperative settings, an expected effect size of Cohen’s d = 0.90 was assumed for postoperative pain reduction [18]. With a two-sided alpha level of 0.05 and a statistical power of 90% (1–β = 0.90), a minimum of 28 participants per group was required, yielding a total sample size of 56 participants. To account for potential attrition and unforeseen exclusions, the target sample size was increased to 35 participants per group (final *N* = 70). No loss to follow-up occurred during the study, resulting in complete outcome data for all randomised participants and an achieved statistical power exceeding the a priori estimate.

### Randomisation and allocation concealment

Participants were randomised in a 1:1 ratio to either the Progressive Relaxation Exercises plus standard care group or the standard care alone group. Randomisation was performed using a computer-generated block randomisation sequence with a fixed block size of six, prepared by an independent statistician who had no involvement in participant recruitment, intervention delivery, or outcome assessment [19]. To minimise the risk of selection bias and allocation predictability, the randomisation sequence was concealed from all clinical and research staff involved in enrolment. Allocation concealment was ensured using sequentially numbered, opaque, sealed envelopes prepared off-site. Envelopes were opened only after completion of baseline assessments and confirmation of eligibility. Group assignment was implemented by a nurse who was not involved in data collection, intervention delivery, or statistical analysis, thereby preserving concealment throughout the enrolment process.

### Blinding

Due to the behavioural nature of the intervention, blinding of participants was not feasible. However, outcome assessors responsible for collecting pain, anxiety, and physiological data were blinded to group allocation. Physiological parameters were obtained using standardised digital monitoring systems by blinded nursing staff, and data entry and statistical analyses were conducted by an independent biostatistician who was unaware of allocation codes. Procedures were in place to maintain blinding integrity, and no protocol deviations related to unblinding were identified during the study period.

### Intervention: progressive relaxation exercises (PRE)

The Progressive Relaxation Exercises (PRE) protocol followed a validated instructional framework based on established PRE principles involving sequential muscle contraction and relaxation, and was delivered exclusively during the postoperative period after confirmation of haemodynamic stability [6]. Permission to use the standardised instructional video was obtained from the Turkish Psychologists Association (12 July 2021). The intervention consisted of three standardised 30-min sessions administered individually at the bedside at postoperative hour 6, postoperative day 1, and postoperative day 2. The timing was selected to target the early postoperative phase characterised by heightened physiological arousal and pain–anxiety interaction.

All sessions were delivered by a nurse trained in PRE using a standardised audio-visual script to ensure consistency across participants. Each session included diaphragmatic breathing, sequential contraction and relaxation of major muscle groups, and a brief integration phase aimed at reinforcing somatic awareness and autonomic downregulation. Environmental conditions, patient positioning, pacing, and verbal cues were standardised for all sessions to maintain intervention fidelity. Adherence was monitored using a structured fidelity checklist, and all participants allocated to the intervention group completed all three sessions as scheduled, resulting in 100% adherence.

### Control group

Participants allocated to the control group received standard postoperative care in accordance with institutional protocols. Standard care included routine nursing care, analgesic administration, vital sign monitoring, oxygen therapy when clinically indicated, and postoperative mobilisation as tolerated. No placebo intervention or attention-control procedure was applied in the control group to avoid interference with routine clinical workflows and to reflect real-world postoperative practice [13].

### Standardised analgesic protocol

To minimise medication-related confounding, all participants followed a standardised postoperative analgesic regimen consistent with multimodal, opioid-sparing principles of postoperative pain management [15].

The protocol comprised intravenous paracetamol 1 g every 8 h as baseline analgesia; non-steroidal anti-inflammatory drugs (tenoxicam 20 mg IV or dexketoprofen 50 mg IV) administered every 12 h as required; and intravenous tramadol 50 mg as rescue analgesia, with a maximum daily dose of 200 mg. Patient-controlled analgesia, epidural analgesia, regional nerve blocks, and sedative agents were not employed during the study period. Analgesic administration was documented using routine medication administration records and quantified at predefined assessment points (postoperative hour 6 and postoperative day 1). These time points were selected to coincide with early postoperative symptom assessment and to ensure consistency between analgesic exposure and outcome measurements.

### Outcome measures

#### Pain assessment

Postoperative pain was assessed using the Short-Form McGill Pain Questionnaire (SF-MPQ), which captures sensory and affective pain descriptors, Present Pain Intensity, and overall pain severity. The SF-MPQ is a widely validated multidimensional pain instrument with established reliability in surgical populations [10]. Pain assessments were conducted preoperatively at baseline and at postoperative hour 6 and postoperative day 2 to capture both early and evolving postoperative pain experiences.

#### Anxiety assessment

Anxiety was measured using the State–Trait Anxiety Inventory (STAI). The STAI-State subscale assesses situational anxiety related to acute stress, whereas the STAI-Trait subscale evaluates dispositional anxiety [11]. STAI-State was assessed at postoperative hour 6 and postoperative day 2, while STAI-Trait was assessed at baseline prior to randomisation and again on postoperative day 2. Higher scores indicate greater anxiety.

#### Physiological parameters

Physiological outcomes included systolic and diastolic blood pressure, heart rate, respiratory rate, and peripheral oxygen saturation, which are commonly used indicators for monitoring clinical status and recovery in surgical patients [20]. Measurements were obtained by blinded nursing staff using standardised digital monitoring systems and recorded at postoperative hour 6, postoperative day 1, and postoperative day 2, corresponding to periods of heightened autonomic activation and early recovery.

#### Pain–anxiety relationship

Associations between postoperative pain outcomes and anxiety scores at postoperative day 2 were examined using Pearson correlation analysis to explore the relationship between nociceptive and psychological responses during recovery.

### Data quality and missing data handling

Data quality was ensured through double-entry verification and cross-checking of all outcome variables against original source records. Prior to analysis, data were screened for accuracy, completeness, and plausibility. Outliers were evaluated using boxplots, standardised z-scores, and Mahalanobis distance; no data points met predefined criteria for exclusion. No missing data were observed for any outcome variable, and complete-case analysis was therefore applied without the need for imputation procedures [21].

### Statistical analysis

Statistical analyses were performed using SPSS version 25. The distribution of continuous variables was assessed for normality using the Shapiro–Wilk test, supported by visual inspection of histograms and Q–Q plots. All primary outcome variables met normality assumptions. Accordingly, parametric tests were applied throughout the analysis [22].

Between-group comparisons for continuous variables were conducted using independent-samples t-tests, while within-group changes over time were examined using paired t-tests. Categorical variables were analysed using the chi-square test. The primary outcome was predefined as the total SF-MPQ score at postoperative day 2; baseline and postoperative hour 6 assessments were used to characterise early pain trajectories and to support covariate adjustment. Postoperative hour 6 pain scores were analysed descriptively to characterise early pain trajectories and were not treated as primary or baseline outcomes. Baseline pain was defined as the preoperative SF-MPQ total score obtained prior to randomisation. Postoperative hour 6 pain was used to characterise early trajectories and was not treated as baseline. To account for potential baseline imbalances, sensitivity analyses were conducted using analysis of covariance (ANCOVA). Covariates included chronic disease status, continuous medication use, baseline pain score, baseline anxiety as measured by the STAI-Trait scale, and pain-coping strategies. Given the exploratory nature of secondary outcomes and the relatively small sample size, p-values for secondary endpoints were interpreted descriptively, with emphasis placed on effect sizes and consistency across outcomes rather than formal adjustment for multiple comparisons [23]. Effect sizes were calculated using Cohen’s d, with thresholds of 0.2 indicating small, 0.5 medium, 0.8 large, and 1.2 very large effects. All statistical tests were two-sided, and a *p*-value < 0.05 was considered statistically significant.

### Ethical considerations

The study was approved by the Institutional Scientific Research Ethics Committee (Approval No: 64). Additional institutional permissions required for conducting the study were obtained from the relevant hospital and health authorities. Permission to use the Progressive Relaxation Exercises instructional video was obtained from the Turkish Psychologists Association. Written informed consent was obtained from all participants prior to enrolment. All study procedures were conducted in accordance with the principles of the Declaration of Helsinki [24].

## Results

### Data screening and assumption checks

Prior to hypothesis testing, all continuous variables were screened for distributional assumptions. Normality was assessed using the Shapiro–Wilk test for all primary and secondary outcome measures, with all tests yielding non-significant results (*p* > 0.05), indicating no violation of normality assumptions. Visual inspection of histograms and Q–Q plots further supported these findings, demonstrating symmetric distributions without marked skewness or kurtosis.Accordingly, parametric statistical analyses were deemed appropriate and applied consistently across all outcome domains. No missing data were identified for any variable at any assessment point, and all 70 randomised participants were retained in the final analyses. The absence of missing data and complete follow-up eliminated the risk of attrition bias and strengthened the internal validity of the findings.

### Participant flow

During the study period, a total of 82 patients undergoing emergency abdominal surgery were assessed for eligibility. Of these, 12 patients were excluded based on predefined exclusion criteria, including cognitive impairment (*n* = 4), postoperative admission to the intensive care unit (*n* = 3), sedative drug administration interfering with participation (*n* = 2), and refusal to participate (*n* = 3).

Seventy eligible participants were subsequently randomised in a 1:1 allocation ratio to either the PRE intervention group (*n* = 35) or the standard care control group (*n* = 35). All randomised participants received their allocated intervention and completed all scheduled outcome assessments at postoperative hour 6, postoperative day 1, and postoperative day 2. No protocol deviations or losses to follow-up were recorded.

**Fig. 1:**
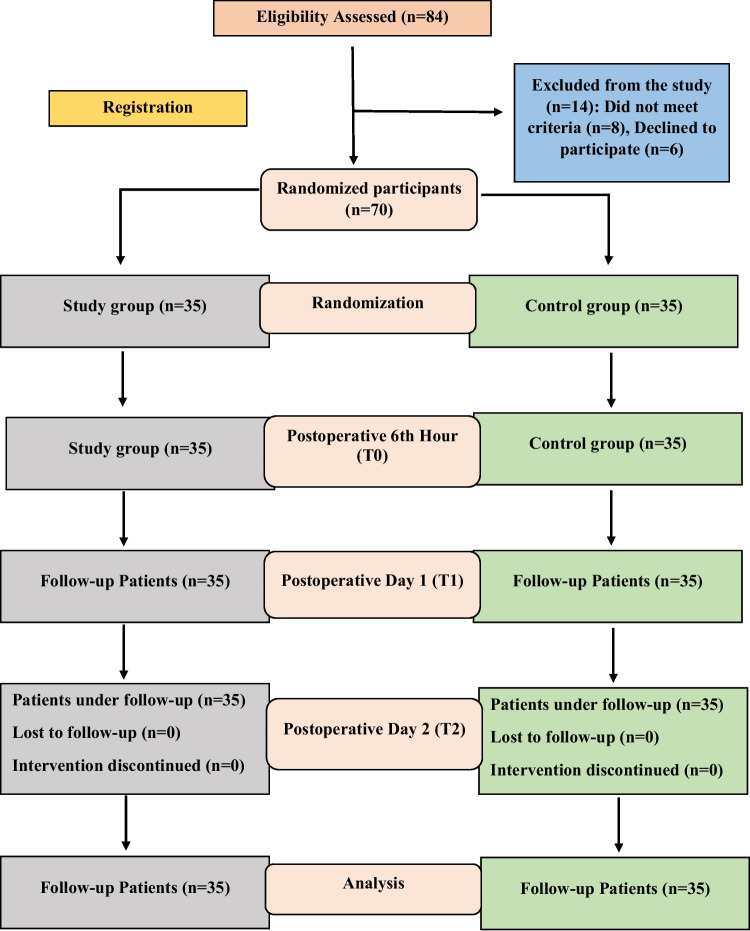
CONSORT flow diagram of participant enrollment, allocation, follow-up, and analysis

Participant enrolment, allocation, follow-up, and inclusion in the final analysis are presented in the CONSORT flow diagram (Fig. 1), demonstrating full adherence to CONSORT reporting standards [17].

### Baseline characteristics

Baseline demographic, clinical, and psychological characteristics were comparable between the Progressive Relaxation Exercises (PRE) and control groups, confirming successful randomisation **(**Table 1).

**Table 1:** Baseline demographic, lifestyle, and clinical characteristics of the study participants (*N* = 70)

Variable	PRE group (*n* = 35)	Control group (*n* = 35)	*p*-value
Age (years)	41.31 ± 17.45	41.28 ± 15.28	0.994
Female sex, *n* (%)	16 (45.7)	13 (37.1)	0.467
Body mass index (kg/m^2^)	26.68 ± 3.55	25.97 ± 3.93	0.425
Current smoking, *n* (%)	15 (42.9)	21 (60.0)	0.151
Previous surgery, *n* (%)	12 (34.3)	8 (22.9)	0.290
Duration of surgery (min)	96.42 ± 36.25	95.57 ± 34.53	0.920
Preoperative STAI-S	46.11 ± 8.62	45.14 ± 7.64	0.673
Preoperative STAI-T	43.28 ± 4.76	44.85 ± 4.52	0.203
Preoperative SF-MPQ total	34.01 ± 7.40	32.04 ± 6.43	0.560
Pain-coping strategies, *n* (%)	26 (74.3)	33 (94.3)	0.022*

Data are presented as mean ± SD or *n* (%). Between-group comparisons were performed using independent-samples t-test or χ^2^ test as appropriate. *PRE* progressive relaxation exercises, *STAI-S* State anxiety ınventory, *STAI-T* trait anxiety ınventory, *SF-MPQ* short-form McGill pain questionnaire. *p* < 0.05

There were no significant differences in age, sex, body mass index, smoking status, surgical duration, or preoperative anxiety and pain scores (all *p* > 0.05).

The only significant difference was found in the prevalence of pain-coping strategies, which was higher in the control group (94.3%) compared with the PRE group (74.3%) (*p* = 0.022); this variable was therefore controlled as a covariate in subsequent ANCOVA analyses.

### Surgical characteristics

The distribution of emergency abdominal surgery types was comparable between the PRE and control groups, with no statistically significant differences observed (all *p* > 0.05; Table 2).

**Table 2:** Distribution of emergency abdominal surgery types between study groups (*N* = 70)

Surgery type	PRE group (*n* = 35)	Control group (*n* = 35)	*p*-value
Appendectomy	16 (45.7)	15 (42.9)	0.812
Small bowel obstruction	7 (20.0)	6 (17.1)	0.764
Perforated viscus	5 (14.3)	6 (17.1)	0.744
Acute cholecystitis	4 (11.4)	5 (14.3)	0.713
Other abdominal procedures	3 (8.6)	3 (8.6)	1.000

Data are presented as n (%). Between-group comparisons were performed using the χ^2^ test

Procedures included appendectomy, small bowel obstruction, perforated viscus repair, and acute cholecystitis, reflecting a representative emergency general surgery cohort.

### Postoperative pain outcomes

At postoperative hour 6, pain scores were similar across groups (all p > 0.05).

By postoperative day 2, the PRE group demonstrated significantly lower sensory pain, pain severity, and total pain scores compared with controls (all *p* ≤ 0.018; Table 3).

**Table 3:** Postoperative pain scores (SF-MPQ) at postoperative hour 6 and day 2

Pain dimension	Time	PRE mean ± SD	Control mean ± SD	Mean difference (95% CI)	*p*-value	Cohen’s *d*
Sensory Pain	6th hour	16.91 ± 4.76	16.14 ± 4.64	0.77 (–1.50, 3.04)	0.642	0.16
	Day 2	10.92 ± 3.56	14.78 ± 4.62	–3.86 (–6.24, –1.48)	0.002*	**0.97**
Pain Severity	6th hour	7.14 ± 1.89	6.51 ± 1.48	0.63 (–0.55, 1.81)	0.371	0.35
	Day 2	3.42 ± 1.65	5.70 ± 1.08	–2.28 (–3.12, –1.44)	0.018*	**1.23**
Present Pain Intensity	Day 2	1.31 ± 0.63	2.00 ± 0.93	–0.69 (–1.12, –0.26)	0.002*	**0.90**
Total Pain Score	Day 2	20.67 ± 5.55	28.34 ± 5.87	–7.67 (–10.35, –4.99)	< 0.001*	**1.51**

Data are presented as mean ± SD. Independent-samples t-test used; 95% CI and Cohen’s d reported. *p* < 0.05 indicates statistical significance

Effect sizes were large to very large (Cohen’s d = 0.96–1.51).

95% CIs confirmed clinically meaningful reductions: for total SF-MPQ score, mean difference –7.67 (95% CI [–10.35, –4.99], *p* < 0.001).

### Anxiety outcomes

No significant between-group difference was observed in STAI-S at postoperative hour 6 (*p* = 0.238).

By postoperative day 2, however, both state and trait anxiety were significantly lower in the PRE group (*p* = 0.004 each; Table 4).

**Table 4:** State and trait anxiety scores (STAI) at postoperative hour 6 and day 2

Scale	Time point	PRE mean ± SD	Control mean ± SD	Mean difference (95% CI)	*p*-value	Cohen’s *d*
STAI-S	Hour 6	46.11 ± 8.62	45.14 ± 7.64	0.97 (–2.79, 4.73)	0.238	0.12
	Day 2	34.00 ± 8.75	41.78 ± 6.63	–7.78 (–11.48, –4.08)	0.004*	**0.85**
STAI-T	Baseline	43.28 ± 4.76	44.85 ± 4.52	–1.57 (–4.58, 1.44)	0.203	0.23
	Day 2	35.21 ± 3.56	42.73 ± 4.32	–7.52 (–9.41, –5.63)	0.004*	**1.05**

Independent-samples t-test; 95% CI and Cohen’s d reported. *STAI-S* State anxiety, *STAI-T* trait anxiety, *PRE* progressive relaxation exercises. *p* < 0.05

Mean differences were –7.78 (95% CI [–11.48, –4.08]) for STAI-S and –7.52 (95% CI [–9.41, –5.63]) for STAI-T, both representing large effect sizes (d = 0.85–1.05).

### Physiological parameters

Participants allocated to the Progressive Relaxation Exercises (PRE) group exhibited more favourable physiological responses during the early postoperative period compared with controls (Table 5). At postoperative hour 6, systolic and diastolic blood pressure values were slightly higher in the PRE group, whereas heart rate and respiratory rate were lower and peripheral oxygen saturation (SpO₂) was higher. Several of these between-group differences persisted on postoperative day 1 but were no longer observed by postoperative day 2 (*p* > 0.05). Overall, these findings indicate more favourable early physiological parameters in the PRE group during the immediate postoperative recovery period.

**Table 5:** Postoperative physiological parameters in the Progressive Relaxation Exercises (PRE) and control groups (*N* = 70)

Time point	Parameter	PRE mean ± SD	Control mean ± SD	Mean difference (95% CI)	*p*-value	Cohen’s *d*
Postop 6 h	SBP (mmHg)	119.20 ± 8.06	117.00 ± 10.06	2.20 (0.07, 4.33)	0.043*	0.45
	DBP (mmHg)	74.28 ± 4.06	71.80 ± 10.13	2.48 (0.41, 4.55)	0.021*	0.54
	HR (bpm)	76.69 ± 7.06	78.72 ± 13.06	–2.03 (–4.01, –0.05)	0.042*	0.50
	RR (bpm)	15.69 ± 3.06	18.72 ± 3.12	–3.03 (–4.01, –2.05)	0.042*	0.68
	SpO₂ (%)	97.35 ± 4.09	95.78 ± 5.13	1.57 (0.42, 2.72)	0.002*	0.79
Postop Day 1	SBP (mmHg)	118.63 ± 8.47	119.10 ± 11.47	–0.47 (–3.52, 2.58)	0.686	0.09
	DBP (mmHg)	79.63 ± 8.47	78.60 ± 10.43	1.03 (0.02, 2.04)	0.048*	0.43
	HR (bpm)	78.90 ± 9.46	80.76 ± 12.49	–1.86 (–3.32, –0.40)	0.028*	0.40
	RR (bpm)	14.23 ± 1.42	15.76 ± 2.42	–1.53 (–2.64, –0.42)	0.023*	0.63
	SpO₂ (%)	98.03 ± 2.43	97.86 ± 2.44	0.17 (0.01, 0.33)	0.042*	0.37
Postop Day 2	SBP (mmHg)	119.71 ± 7.47	120.00 ± 8.04	–0.29 (–1.92, 1.34)	0.642	0.06
	DBP (mmHg)	79.01 ± 6.47	79.06 ± 6.04	–0.05 (–0.75, 0.65)	0.742	0.01
	HR (bpm)	80.03 ± 7.47	80.96 ± 10.04	–0.93 (–2.24, 0.38)	0.099	0.22
	RR (bpm)	15.01 ± 1.27	15.86 ± 1.06	–0.85 (–1.96, 0.26)	0.663	0.18
	SpO₂ (%)	98.71 ± 1.37	97.69 ± 1.74	1.02 (–0.09, 2.13)	0.153	0.31

Independent-samples t-test. 95% CI and Cohen’s d reported. *SBP* systolic blood pressure, *DBP* diastolic blood pressure, *HR* heart rate, *RR* respiratory rate, *SpO₂* peripheral oxygen saturation, *PRE* progressive relaxation exercises. *p* < 0.05 indicates significance

### Analgesic use

There were no significant between-group differences in postoperative analgesic consumption (Table 6).

**Table 6:** Postoperative analgesic consumption in the progressive relaxation exercises (PRE) and control groups

Analgesic	Time point	PRE mean ± SD	Control mean ± SD	Mean difference (95% CI)	*p*-value	Cohen’s *d*
NSAID	6th hour	2.02 ± 0.51	2.11 ± 0.47	–0.09 (–0.34, 0.16)	0.469	0.18
	Day 1	1.68 ± 0.52	1.85 ± 0.42	–0.17 (–0.42, 0.08)	0.142	0.34
Opioid (Tramadol)	Day 1	0.95 ± 0.50	1.05 ± 0.41	–0.10 (–0.35, 0.15)	0.443	0.21

Data are mean ± SD. Independent-samples t-test. 95% CI and Cohen’s d reported. *NSAID* nonsteroidal anti-inflammatory drug, *PRE* progressive relaxation exercises

At postoperative hour 6, NSAID use did not differ significantly between the PRE and control groups (mean difference = –0.09, 95% CI [–0.34, 0.16], *p* = 0.469).

Similarly, no differences were observed for NSAID or opioid (tramadol) consumption on postoperative day 1 (*p* = 0.142 and *p* = 0.443, respectively).

These findings indicate that observed improvements in pain and anxiety outcomes were not attributable to differential pharmacological exposure.

### Pain–anxiety relationship

Moderate positive correlations were observed between postoperative pain scores and anxiety at postoperative day 2 (Table 7).

**Table 7:** Correlations between postoperative pain outcomes and anxiety at postoperative day 2

Pain outcome (SF-MPQ)	PRE group (*n* = 35) *r* (95% CI)	*p*-value	Control group (*n* = 35) *r* (95% CI)	*p*-value
Sensory pain score	0.436 (0.12, 0.68)	0.009*	0.293 (0.02, 0.53)	0.032*
Present Pain Intensity (PPI)	0.395 (0.09, 0.63)	0.012*	0.248 (0.01, 0.47)	0.039*
Total SF-MPQ score	0.426 (0.11, 0.67)	0.009*	0.265 (0.01, 0.49)	0.036*

Pearson correlation coefficients (r) are presented with 95% confidence intervals. Anxiety measured using the State–Trait Anxiety Inventory (STAI). *SF-MPQ* Short-form McGill pain questionnaire, *PPI* present pain ıntensity, *PRE* progressive relaxation exercises. *p* < 0.05 significant

In the PRE group, sensory pain (r = 0.436, 95% CI [0.12, 0.68], *p* = 0.009), Present Pain Intensity (r = 0.395, 95% CI [0.09, 0.63], *p* = 0.012), and total SF-MPQ score (r = 0.426, 95% CI [0.11, 0.67], *p* = 0.009) correlated moderately with anxiety.

Similar but weaker correlations were observed in the control group (r = 0.248–0.293, all *p* < 0.05).

These findings support a bidirectional pain–anxiety amplification model, wherein increased anxiety heightens pain perception and greater pain further intensifies anxiety during early postoperative recovery.

### ANCOVA-adjusted outcomes

To control for potential baseline imbalances including chronic disease status, continuous medication use, baseline pain and anxiety scores, and pain-coping strategies analysis of covariance (ANCOVA) was performed for all primary outcomes. After adjustment, PRE remained significantly superior across all major domains.

For pain outcomes, adjusted models confirmed significantly lower sensory pain (F(1, 66) = 14.92, *p* < 0.001, partial η^2^ = 0.18), pain severity (F(1, 66) = 10.40, *p* = 0.002, partial η^2^ = 0.14), general pain intensity (F(1, 66) = 11.27, *p* = 0.001, partial η^2^ = 0.15), and total SF-MPQ scores (F(1, 66) = 19.88, *p* < 0.001, partial η^2^ = 0.23) in the PRE group compared with controls.

For anxiety, PRE participants demonstrated significantly lower adjusted STAI-S (F(1, 66) = 9.14, *p* = 0.003, partial η^2^ = 0.12) and STAI-T (F(1, 66) = 12.51, *p* = 0.001, partial η^2^ = 0.16) scores.

These adjusted results remained consistent with unadjusted analyses, confirming the robustness of PRE’s effect independent of baseline covariates. Collectively, ANCOVA results reinforce that the analgesic, anxiolytic, and physiological benefits of PRE reflect genuine psychophysiological improvements rather than artefacts of sample variation or baseline imbalances.

## Discussion

This randomized controlled trial found that Progressive Relaxation Exercises (PRE), when delivered exclusively during the early postoperative period, were associated with significant reductions in pain and anxiety together with improvements in physiological stability among patients undergoing emergency abdominal surgery. These findings suggest that PRE may serve as a beneficial adjunct to multimodal postoperative care by modulating both psychological and physiological stress responses. Given the high prevalence of psychological distress among surgical patients, particularly the substantial rates of preoperative anxiety reported to exceed 50% among hospitalized individuals, the observed anxiolytic effects of PRE highlight its potential clinical relevance as a feasible and effective strategy for perioperative anxiety management within comprehensive surgical care pathways [25].

### Postoperative pain

Patients who received PRE experienced significantly lower postoperative pain scores by the second postoperative day, with large effect sizes (Cohen’s d = 1.51, *p* < 0.001). The magnitude of pain reduction in this study closely parallels findings from other randomized controlled trials showing that relaxation-based interventions substantially alleviate pain following major surgery [26–29]. Akıncı et al. [26] reported significantly decreased pain intensity and earlier mobilization among laparoscopic kidney donors receiving PRE, while Mashhadi-Naser and Shirvani [27] and Bagheri-Mottahedi et al. [28] documented similar benefits across orthopedic and general surgical cohorts. A meta-analysis confirmed that structured relaxation reduces postoperative pain in abdominal surgery, further supporting its role as a non-pharmacologic analgesic adjunct [30].

The temporal pattern observed in this study, with minimal difference at six hours followed by substantial reduction by day two, suggests that PRE exerts its effect during the transition from acute sympathetic activation to autonomic recovery. This mechanism aligns with evidence that relaxation enhances parasympathetic tone, attenuates stress-related endocrine activity, and decreases central sensitization [27, 30]. Consequently, PRE appears to modulate the affective dimension of pain rather than direct nociceptive signaling, providing clinically meaningful pain relief without altering pharmacologic management.

### Postoperative anxiety

Both state and trait anxiety scores decreased significantly by postoperative day two, with mean reductions of 7.78 and 7.52 points, respectively. These results are consistent with prior randomized trials reporting that relaxation-based interventions effectively reduce perioperative anxiety and stress [31–33]. Akıncı et al. [32] observed similar anxiety improvements in living kidney donors, while Mashhadi-Naser and Shirvani [31] confirmed significant decreases in both anxiety subscales in orthopedic surgery patients. Comparable anxiolytic effects have also been demonstrated across various clinical populations, indicating that even brief PRE sessions can produce clinically relevant reductions in psychological distress [34, 35].

The anxiolytic mechanisms of PRE are primarily associated with vagal activation and suppression of hypothalamic–pituitary–adrenal axis hyperactivity, both of which restore autonomic balance disrupted by surgical stress. Previous studies have shown that relaxation training reduces heart rate and respiratory rate while improving emotional regulation [34, 35]. The transient decline in trait anxiety observed in this study likely reflects short-term normalization of cognitive appraisal rather than a stable personality shift, consistent with other postoperative trials [31, 33]. Together, these findings emphasize that PRE simultaneously targets both emotional and physiological dimensions of recovery, facilitating adaptation to postoperative stress.

### Physiological stability and autonomic regulation

In addition to psychological outcomes, this study demonstrated favourable early postoperative physiological changes. At postoperative hour 6, systolic and diastolic blood pressure values were modestly higher in the PRE group, while heart rate and respiratory rate were lower and oxygen saturation was higher, suggesting early autonomic modulation during peak postoperative stress. Despite these modest differences in blood pressure, the concurrent reductions in heart and respiratory rates together with improved oxygen saturation indicate a pattern of early autonomic modulation characterised by reduced cardiopulmonary workload. Importantly, the modest between-group differences in blood pressure were small in absolute magnitude and occurred alongside lower heart and respiratory rates and higher oxygen saturation, supporting improved autonomic efficiency during early recovery rather than clinically meaningful haemodynamic strain. These findings corroborate prior clinical evidence showing that relaxation-based interventions promote short-term cardiovascular and respiratory equilibrium [36–38]. Kömürkara and Cengiz [36] reported similar improvements in vital signs among liver-transplant patients, while Korkut and Oğuzhan [37] observed favourable haemodynamic and anxiety-related effects following brief bedside PRE sessions. Collectively, these observations align with the concept of relaxation-induced parasympathetic rebound during acute stress recovery.

The transient nature of these physiological changes prominent in the first 24 h and resolving by day two suggests that PRE facilitates early autonomic realignment rather than persistent hemodynamic alteration. Consistent with previous research, parasympathetic activation appears temporarily to override sympathetic dominance, leading to reduced cardiopulmonary workload and improved oxygenation [39, 40]. This physiological stabilization complements the observed psychological benefits, indicating that PRE supports recovery through integrated modulation of both autonomic and affective systems.

### Pain–anxiety relationship and analgesic use

Although PRE significantly reduced pain and anxiety, postoperative analgesic consumption did not differ between groups, suggesting that these improvements were achieved independently of pharmacologic intervention. This outcome is consistent with evidence showing that mind–body interventions enhance pain and stress management without increasing or decreasing analgesic intake [41–43]. Nompo Fatimah et al. [41] reported significant reductions in pain and anxiety following relaxation-based programs despite stable medication use, while Olbrecht and Williams [42] found similar results in postoperative patients receiving guided relaxation under multimodal analgesia protocols.

Moderate positive correlations between pain and anxiety outcomes (r = 0.395–0.436, *p* < 0.05) in this study support the bidirectional amplification model, wherein heightened anxiety intensifies pain perception and vice versa. This dynamic relationship has been confirmed in multiple perioperative studies, emphasizing the need for integrated interventions that address both dimensions concurrently [8, 43, 44]. PRE may disrupt this cycle by downregulating sympathetic activation and promoting affective recalibration, as shown in previous clinical and experimental studies [8, 44]. These findings reinforce the psychophysiological foundation of PRE and highlight its practical utility as a nurse-led, non-invasive strategy to enhance postoperative recovery in high-stress surgical environments.

### Clinical integration and broader implications

From an implementation perspective, the practical attributes of PRE, including its brevity, bedside applicability, and nurse-led delivery model, make it particularly well suited to the demanding environment of emergency surgical wards, where opportunities for structured patient education and preoperative psychological preparation are inherently limited. Within this context, PRE aligns closely with the patient-centred philosophy of Enhanced Recovery After Surgery (ERAS) pathways, addressing both physiological and psychological aspects of recovery through a non-invasive, low-cost, and easily scalable intervention.

Taken together, the findings of this trial indicate that nurse-delivered Progressive Relaxation Exercises yield clinically meaningful improvements in pain, anxiety, and early postoperative physiological stability. The intervention’s multidimensional benefits, achieved without modification of pharmacological care, underscore its potential as an effective behavioural adjunct within integrated, ERAS-oriented perioperative recovery frameworks.

## Conclusion

This randomized controlled trial provides the first evidence that Progressive Relaxation Exercises delivered exclusively during the postoperative period are effective in patients undergoing emergency abdominal surgery. By producing significant reductions in postoperative pain and anxiety within the first 48 h after surgery, this study provides novel evidence supporting the use of *postoperative-only* relaxation interventions in high-acuity emergency surgical settings.

Importantly, the observed benefits of PRE occurred independently of pharmacological analgesic consumption, indicating that improvements in pain and anxiety were achieved without escalating drug therapy. This finding is particularly relevant in emergency surgery, where analgesic optimisation is often constrained and non-pharmacological strategies are needed to support recovery. The associated improvements in early physiological stability further suggest that PRE facilitates beneficial autonomic regulation during the period of peak postoperative stress.

From a clinical perspective, PRE are a simple, low-cost, nurse-led intervention that can be delivered at the bedside without additional equipment or specialist resources. Its feasibility and scalability make it directly relevant to surgeons, nurses, and multidisciplinary teams working in emergency surgical units. Integrating PRE into routine postoperative care may enhance patient-centred recovery and complement ERAS-aligned perioperative pathways, with direct relevance for surgeons, nurses, and multidisciplinary teams working in emergency surgical units.

## Strengths and limitations

### Strengths

This study has several notable strengths. It was conducted as a rigorously designed randomised controlled trial in full accordance with CONSORT 2010 guidelines, incorporating robust methodological safeguards including block randomisation, strict allocation concealment using sequentially numbered opaque sealed envelopes, blinded outcome assessment, and prospective trial registration (ClinicalTrials.gov: NCT07301073). These measures collectively minimised selection, detection, and performance bias.

A key strength lies in the clinically novel application of Progressive Relaxation Exercises delivered exclusively *during the postoperative period,* addressing a clear evidence gap that is particularly relevant in emergency surgery, where preoperative psychological interventions are often not feasible. The inclusion of emergency abdominal surgery patients a high-risk and underrepresented population in non-pharmacological research further enhances the clinical relevance of the findings. Additional strengths include the use of internationally validated instruments for pain and anxiety assessment (SF-MPQ and STAI), incorporation of objective physiological parameters, complete data retention without attrition, and consistency between unadjusted and ANCOVA-adjusted analyses, all of which support the internal validity of the trial.

### Limitations

Several limitations should be acknowledged. First, the single-centre design may limit the generalisability of the findings to other healthcare systems or emergency surgical settings. Second, outcomes were assessed only during the early postoperative period, precluding conclusions regarding longer-term recovery or functional outcomes. Third, participant blinding was not feasible due to the behavioural nature of the intervention, although this was partially mitigated through blinded outcome assessment and independent statistical analysis.

However, these limitations are balanced by the pragmatic study design, rigorous methodology, and clinically meaningful effect sizes observed across pain, anxiety, and physiological outcomes.

### Clinical and research implications

The findings indicate that postoperative Progressive Relaxation Exercises constitute a feasible, low-cost, and well-tolerated adjunct to standard postoperative care in emergency surgical settings. The observed reductions in pain and anxiety without changes in analgesic consumption support PRE as a complementary, non-pharmacological strategy aligned with ERAS principles and opioid-sparing approaches. Clinically, PRE can be readily implemented at the bedside by nursing staff without specialised equipment, reinforcing holistic and patient-centred postoperative care. Future research should prioritise multicentre trials with extended follow-up and the inclusion of objective physiological markers to further elucidate mechanisms and support broader integration into contemporary perioperative practice.

## Data Availability

The datasets generated and analysed during the current study are available from the corresponding author upon reasonable request. De-identified participant data, statistical code, and study materials will be shared following publication in accordance with institutional and ethical regulations.

## Declarations

### AI declaration

During the preparation of this manuscript, ChatGPT was used to assist with language editing and structural refinement. All content was critically reviewed, verified, and approved by the authors, who take full responsibility for the integrity and originality of the work.
